# Deleted in liver cancer-1 inhibits cell growth and tumorigenicity in human pancreatic cancer

**DOI:** 10.3892/ol.2013.1415

**Published:** 2013-06-18

**Authors:** ZHENJIANG ZHENG, CHUNLU TAN, GUANGMING XIANG, GANG MAI, XUBAO LIU

**Affiliations:** 1 Department of General Surgery, The Third People’s Hospital of Chengdu, The Second Affiliated Hospital of Chengdu, Chongqing Medical University, Chengdu, Sichuan 610031, P.R. China; 2 Department of Hepatobiliopancreatic Surgery, West China Hospital, Sichuan University, Chengdu, Sichuan 610041, P.R. China

**Keywords:** deleted in liver cancer, tumor suppressor gene, gene therapy, pancreatic cancer

## Abstract

Deleted in liver cancer-1 (*DLC-1*) has been isolated from primary hepatocellular carcinoma and demonstrated to be a potential tumor suppressor gene. The aim of the present study was to observe the effect of the DLC-1 gene on pancreatic cancer cell growth and evaluate the feasibility of using the DLC-1 gene in gene therapy for pancreatic cancer. A recombinant plasmid (pcDNA3.1/DLC-1) was transfected into PANC-1 cells by liposomes and then the pre-established human PANC-1 pancreatic carcinoma cells were injected into athymic nude mice via the tail vein. The results showed that the overexpression of DLC-1 in the PANC-1 cells inhibited cell proliferation *in vitro*, while the act of introducing DLC-1 reduced tumorigenicity in the nude mice. The findings suggest that DLC-1 may have an effect on the pathogenesis of pancreatic cancer. The DLC-1 gene may be a promising target in gene therapy for pancreatic cancer.

## Introduction

Deleted in liver cancer-1 (*DLC-1*) is a potential tumor suppressor gene, which has been isolated from human hepatocellular carcinoma and identified by representational difference analysis. DLC-1 is localized on human chromo-some 8p21.3-22. The full-length cDNA for DLC-1 contains 3,800 bp and encodes a 1,091-amino acid protein that has 86% homology with the rat pl22RhoGAP gene (1).

DLC-1 (also known as ARHGAP7 and STARD12) contains three functional domains: The RhoGTPase-activating protein (RhoGAP) domain, the steroidogenic acute regulatory-related lipid transfer (START) domain and the sterile α-motif (SAM) domain (2,3). Studies have demonstrated that the RhoGAP domain is necessary for inhibiting tumor cell growth, as well as for actin fiber and focal adhesion formation (4–6). RhoGAPs negatively regulate the Rho family of small GTPases, enhancing the hydrolysis of bound GTP to convert Rho proteins to their inactive GDP-bound state (7,8).

DLC-1 mRNA is expressed in the majority of normal human tissues and is downregulated or absent in a number of common types of human cancer, including brain, lung, breast, liver, stomach, colon and prostate cancers. The aberrant expression of DLC-1 is associated with either genomic deletion or promoter hypermethylation (9–14). Increasing evidence has shown that DLC-1 negatively regulates tumor cell growth and *in vivo* tumorigenicity (15–17).

However, DLC-1 has been less intensively examined in pancreatic cancer. To obtain further evidence that DLC-1 functions as a tumor suppressor gene, in the present study, a recombinant plasmid (pcDNA3.1/DLC-1) was constructed and transduced into PANC-1 cells, in order to observe the effect of the DLC-1 gene on cell growth and tumorigenicity.

## Materials and methods

### Cell line and culture

The human pancreatic carcinoma cell line, PANC-1, was obtained from the Shanghai Institute of Biochemistry and Cell Biology (Chinese Academy of Sciences, Shanghai, China). The cells were cultured in Dulbecco’s modified Eagle’s medium (DMEM) supplemented with 10% fetal bovine serum (FBS) and antibiotics at 37°C, in a humidified incubator with 5% CO_2_.

### Plasmid construction

A 3.4-kb fragment of the full-length coding sequence of the DLC-1 gene was amplified by PCR from human liver PCR-Ready cDNA (Invitrogen, Carlsbad, CA, USA). The primers included *Nhe*I and *Kpn*I linkers. Subsequent to purification and restriction digestion, the PCR product was ligated to the pcDNA3.1(+) vector (Invitrogen). The sequence and orientation of the DLC-1 recombinant were confirmed by DNA sequencing and restriction enzyme digestion.

### Cell transfection

The cells (10^5^) were seeded into 24-well plates one day prior to transfection. The cells were transfected with 1 *μ*g plasmid DNA in Lipofectamine 2000 (Invitrogen), according to the manufacturer’s instructions.

### RNA extraction and RT-PCR

Total RNA was extracted from cells using TRIzol reagent (Invitrogen). The total RNA (2 *μ*g) was used as a template in the first strand cDNA synthesis using a First-Strand cDNA Synthesis kit (Shinegene, Shanghai, China) according to the manufacturer’s instructions. Total RNA (2 *μ*g) was combined with 0.1 *μ*g oligo(dT)_18_ primer and diethylpyrocarbonate (DEPC) H_2_O and preheated at 65°C for 5 min. The mixture was then placed at 20°C for 10 min, then 10 *μ*l 2X First-Strand Buffer and 1 *μ*l RT mix was added for a total volume of 20 *μ*l. The mixture was incubated at 42°C for 50 min, then the reaction was stopped by heating at 90°C for 5 min. The cDNA stock was stored at −20°C. A pair of primers (forward, GGAATAACGGCTCTGTGAA and reverse, TCTCCGACCACTGATTGAC) was used to amplify the 400-bp fragment of DLC-1. As a control, a pair of primers (forward, GTGGACATCCGCAAAGAC and reverse, AAA GGGTGTAACGCAACTAA) was used to amplify the 200-bp fragment of β-actin. PCR was performed using a PTC-200 PCR machine (MJ Research Inc, Waltham, MA, USA). The reaction conditions were as follows: 94°C for 3 min, then 35 cycles of 94°C for 1 min, 55°C for 30 sec and 72°C for 1 min, followed by a final extension step for 10 min at 72°C.

### Western blot analysis

The cells were harvested and solubilized in cold RIPA buffer. Proteins were resolved by SDS-PAGE and transferred to polyvinylidene fluoride (PVDF) membranes. DLC-1 was detected by western blotting using mouse anti-human DLC-1 antibody (BD Biosciences, Franklin Lakes, NJ, USA). β-actin staining served as the internal standard for all membranes.

### MTT assays

The cells were plated in 96-well microtiter plates at a density of 10^4^ cells/well, then cultured for 48 h and incubated with 20 *μ*l MTT solution (5 mg/ml) for 4 h. The cells were lysed in 150 *μ*l DMSO, and the absorbance at 490 nm was determined with an ELISA plate reader. The absorbance values for the cell lines transfected with pcDNA3.1(+) alone, pcDNA3.1(+)/DLC-1 and the untransfected cells were compared. The entire experiment was performed three times independently.

### Preparation of liposome:plasmid complexes

Plasmids were purified using alkaline lysis. Liposomes were composed of 1,2-dioleoyl-3-trimethylammonium-propane and cholesterol in a 1:1 molar ratio, and the dried lipid film was resuspended with 5% dextrose in water. Following the hydration of the lipids, using a bath sonicator, the liposomes were sonicated until clear. The liposomes were then extruded through poly-carbonate membranes and stored at 4°C until use. Prior to injection, the mixture containing liposomes in a 3:1 mass ratio with the plasmid DNA was incubated at room temperature for 20 min.

### Tumorigenicity assay

The PANC-1 cells (10^7^) were inoculated subcutaneously into the right oxter of four-week-old female Balb/c athymic nude mice. Eight days subsequent to the injection of cells, the mice were randomly divided into three groups: i) The liposome:pcDNA3.1(+)/DLC-1 group; ii) the liposome:pcDNA3.1(+) group; and iii) the isosmotic saline treatment group. Each group contained 10 mice and each mouse received seven intravenous injections via the tail vein, five days apart. Each injection (200 *μ*l) consisted of liposomes (150 *μ*g) complexed to 50 *μ*g of a plasmid encoding DLC-1 or a control plasmid. Tumor size was measured in two dimensions prior to each injection and five days subsequent to the last injection, using a vernier caliper. This study was approved by the ethics committee of West China Hospital, Sichuan University, China.

### Statistical analysis

Data are expressed as the mean ± SD. All statistical analyses were performed with standard statistical programs (SPSS for Windows, version 17.0; SPSS, Inc., Chicago, IL, USA). A one-way ANOVA was used for the statistical analysis. P<0.05 was considered to indicate a statistically significant difference.

## Results

### Overexpression of the DLC-1 gene by liposome-mediated transfection

**Figure 1.**
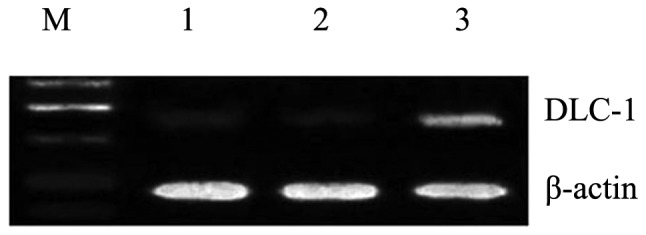
RT-PCR analysis for the presence of the DLC-1 gene in the PANC-1 cell line. M, marker; lane 1, untransfected cells; lane 2, empty vector-transfected cells; lane 3, pcDNA3.1(+)/DLC-1-transfected cell. β-actin was analyzed as a positive control. The expression of mRNA was upregulated in the pcDNA3.1(+)/DLC-1 transfected cells.

**Figure 2.**
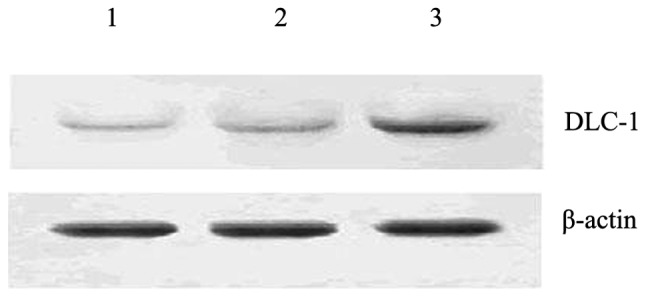
Western blot analysis of DLC-1 protein in the PANC-1 cell line. Lane 1, untransfected cells; lane 2, empty vector-transfected cells; lane 3, pcDNA3.1(+)/DLC-1-transfected cells. Weak bands were detected in the empty vector-transfected and untransfected cells. The molecular size of DLC-1 is 123 kDa; β-actin is 42 kDa.

Subsequent to 48 h of transfection, the transfection of the DLC-1 gene into the PANC-1 cells was detected by semi-quantitative RT-PCR and western blotting, respectively. As shown in Figs. 1 and 2, a successful transfer of DLC-1 by the liposome complex was demonstrated. In the pcDNA3.1(+)/DLC-1-transfected cells, the mRNA and protein expression levels of DLC-1 were upregulated. By contrast, only weak bands were observed in the empty vector-transfected and untransfected cells.

### Overexpression of the DLC-1 gene inhibits cell proliferation

**Figure 3.**
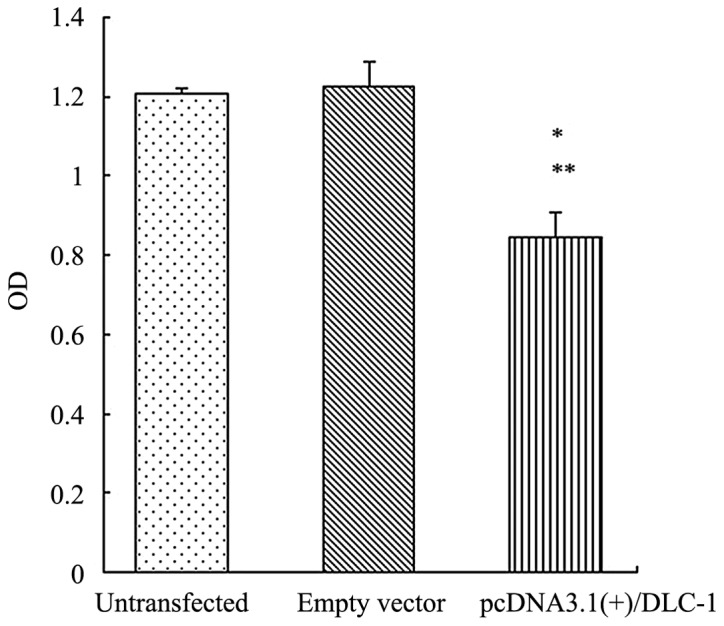
MTT assay for the assessment of cell proliferation in the PANC-1 cell line. OD values were measured in each group (untransfected, empty vector and pcDNA3.1(+)/DLC-1). Significant growth inhibition was observed. ^*^P<0.05 vs. untransfected; ^**^P<0.05 vs. empty vector.

To investigate whether DLC-1 was involved in the cell proliferation of PANC-1 cells, an MTT assay was performed subsequent to 48 h of transfection. As shown in Fig. 3, the OD value was lower in the cells transfected with pcDNA3.1(+)/DLC-1 compared with the cells transfected with the empty vector and the untransfected cells (P<0.05). The results demonstrated that DLC-1 had an effect on cell proliferation.

### Inhibition of in vivo tumorigenicity by the DLC-1 gene

**Figure 4.**
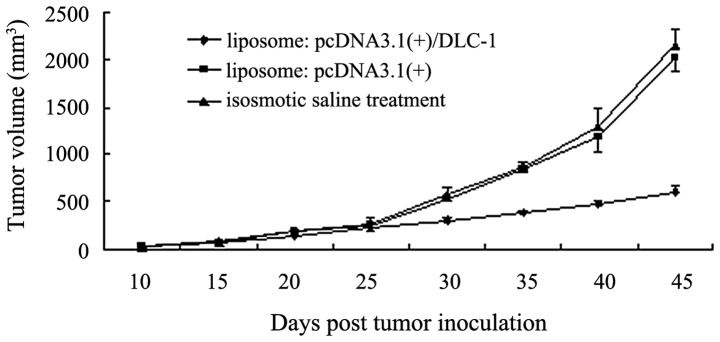
Injection of liposome:DNA complexes into athymic nude mice bearing PANC-1 tumors. Tumor volume (mm^3^) was measured in all groups at each treatment injection time. By the fifth injection, the liposome:pcDNA3.1(+)/DLC-1 was statistically different from the liposome:pcDNA3.1(+) and isosmotic saline treatment groups (^*^P<0.05).

To investigate the effect of DLC-1 on tumor growth, the liposome:DNA complex was injected into athymic nude mice via the tail vein using pre-established human PANC-1 pancreatic carcinoma cells. As shown in Fig. 4, the tumors from the liposome:pcDNA3.1(+)/DLC-1 group were smaller than those from the liposome:pcDNA3.1(+) and isosmotic saline treatment groups after the fifth injection (P<0.05). This result indicates that DLC-1 gene therapy using liposomes as a carrier effectively inhibits tumor growth *in vivo*.

## Discussion

In the United States, 42,470 patients were diagnosed with and 35,240 patients succumbed to pancreatic cancer in 2009 (18). Pancreatic cancer is the fourth most common cause of cancer-related mortality in the United States. Due to the lack of effective screening modalities, the majority of patients are diagnosed with pancreatic cancer at a regional or distant stage of the disease. The overall five-year relative survival rate for patients with pancreatic cancer is 5% (18). At present, surgery is the only curative therapeutic approach. However, only 5 to 25% of patients with pancreatic cancer are suitable for resection at the time of diagnosis (19).

With recent developments in molecular biology techniques and following the mapping of the entire human genome, gene therapy for pancreatic cancer is becoming available. It has been reported that DLC-1 is expressed in a number of normal human tissues and is downregulated or absent in various types of human cancer (9–11). Reduced mRNA levels have also been observed in certain tumor cell lines (15,20,21). These results suggest that DLC-1 may function as a tumor suppressor.

In the present study, the overexpression of the DLC-1 gene in the PANC-1 cell line resulted in the inhibition of cell growth *in vitro*. This result is consistent with other studies. Wong *et al* showed that the overexpression of DLC-1 in SMMC-7721 human HCC cells that lack endogenous DLC-1 expression was able to inhibit cell proliferation and invasiveness (5). In addition, Healy *et al* observed that the restoration of DLC-1 expression in non-small cell lung cancer cell lines impaired anchorage-dependent and -independent growth, as well as invasion *in vitro* (22). It was demonstrated that the suppressive function may be attributable to the biological functions of the DLC-1 gene, which include the organization of the cytoskeleton, the formation of focal adhesions and the induction of apoptosis (4,17,23).

Liposome-mediated intravenous gene delivery in animals usually results in expression in the major organs, including the lung, kidney, spleen and liver, and is not associated with autoimmunity and toxicity (24). Transgenes have continued to be expressed in large numbers of cells in multiple tissues for at least nine weeks without any apparent treatment-related toxicity following a single intravenous injection of liposome:plasmid complexes (25). Chen *et al* noted that i.v. injections of liposomes complexed to PCI-endostatin inhibited the growth of MDA-MB-435 tumors implanted in the mammary fat pads of nude mice by ∼40% compared with either empty vector (PCI) or untreated controls (26).

Thus, this method of gene transfer may be appropriate for human gene therapy. In the present study, it was observed that liposome:DNA complexes administered intravenously decreased the growth of subcutaneously-inoculated tumors, demonstrating that DLC-1 had an effect on tumorigenicity *in vivo*, which is a result supported by other studies (16,17).

In summary, in the present study, the overexpression of the DLC-1 gene in the PANC-1 cells inhibited cell proliferation, suggesting that the DLC-1 gene maybe be a tumor suppressor for pancreatic cancer. The results of this experiment suggested that the DLC-1 gene may be a promising target in gene therapy for pancreatic cancer. The present study also provides experimental evidence for further research into gene therapy for pancreatic cancer.

## References

[1] Yuan BZ, Miller MJ, Keck CL, Zimonjic DB, Thorgeirsson SS, Popescu NC (1998). Cloning, characterization and chromosomal localization of a gene frequently deleted in human liver cancer (DLC-1) homologous to rat RhoGAP. Cancer Res.

[2] Ching YP, Wong CM, Chan SF (2003). Deleted in liver cancer (DLC) 2 encodes a RhoGAP protein with growth suppressor function and is underexpressed in hepatocellular carcinoma. J Biol Chem.

[3] Kawai K, Kiyota M, Seike J, Deki Y, Yagisawa H (2007). STARTGAP3/DLC3 is a GAP for RhoA and Cdc42 and is localized in focal adhesions regulating cell morphology. Biochem Biophys Res Commun.

[4] Sekimata M, Kabuyama Y, Emori Y, Homma Y (1999). Morphological changes and detachment of adherent cells induced by p122, a GTPase-activating protein for Rho. J Biol Chem.

[5] Wong CM, Yam JW, Ching YP (2005). Rho GTPase-activating protein deleted in liver cancer suppresses cell proliferation and invasion in hepatocellular carcinoma. Cancer Res.

[6] Yam JW, Ko FC, Chan CY, Jin DY, Ng IO (2006). Interaction of deleted in liver cancer 1 with tensin2 in caveolae and implications in tumor suppression. Cancer Res.

[7] Moon SY, Zheng Y (2003). Rho GTPase activating proteins in cell regulation. Trends Cell Biol.

[8] Peck J, Douglas G, Wu CH, Burbelo PD (2002). Human RhoGAP domain-containing proteins: structure, function, and evolutionary relationships. FEBS Lett.

[9] Ng IO, Liang ZD, Cao L, Lee TK (2000). DLC-1 is deleted in primary hepatocellular carcinoma and exerts inhibitory effects on the proliferation of hepatoma cell lines with deleted DLC-1. Cancer Res.

[10] Gatalica Z, Velagaleti G, Kuivaniemi H (2005). Gene expression profile of an adenomyoepithelioma of the breast with a reciprocal translocation involving chromosomes 8 and 16. Cancer Genet Cytogenet.

[11] Zhang X, Feng J, Cheng Y (2005). Characterization of differentially expressed genes in ovarian cancer by cDNA microarrays. Int J Gynecol Cancer.

[12] Kim TY, Jong HS, Song SH (2003). Transcriptional silencing of the DLC-1 tumor suppressor gene by epigenetic mechanism in gastric cancer cells. Oncogene.

[13] Healy KD, Kim TY, Shutes AT, Bang YJ, Juliano RL, Der CJ (2006). RhoGAP DLC-1 tumor suppression and aberrant Rho GTPase activation in lung cancer. Proc Am Assoc Cancer Res.

[14] Guan M, Zhou X, Soulitzis N, Spandidos DA, Popescu NC (2006). Aberrant methylation and deacetylation of deleted in liver cancer-1 gene in prostate cancer: potential clinical applications. Clin Cancer Res.

[15] Yuan BZ, Jefferson AM, Baldwin KT, Thorgeirsson SS, Popescu NC, Reynolds SH (2004). DLC-1 operates as a tumor suppressor gene in human non-small cell lung carcinomas. Oncogene.

[16] Yuan BZ, Zhou X, Durkin ME (2003). DLC-1 gene inhibits human breast cancer cell growth and in vivo tumorigenicity. Oncogene.

[17] Zhou X, Thorgeirsson SS, Popescu NC (2004). Restoration of DLC-1 gene expression induces apoptosis and inhibits both cell growth and tumorigenicity in human hepatocellular carcinoma cells. Oncogene.

[18] Jemal A, Siegel R, Ward E, Hao Y, Xu J, Thun MJ (2009). Cancer Statistics, 2009. CA Cancer J Clin.

[19] Neoptolemos JP, Stoken DD, Friess H (2004). A randomized trial of chemoradiotherapy and chemotherapy after resection of pancreatic cancer. N Engl J Med.

[20] Goodison S, Yuan J, Sloan D (2005). The RhoGAP protein DLC-1 functions as a metastasis suppressor in breast cancer cells. Cancer Res.

[21] Wong CM, Lee JM, Ching YP, Jin DY, Ng IO (2003). Genetic and epigenetic alterations of DLC-1 gene in hepatocellular carcinoma. Cancer Res.

[22] Healy KD, Hodgson L, Kim TY (2008). DLC-1 suppresses non-small cell lung cancer growth and invasion by RhoGAP-dependent and independent mechanisms. Mol Carcinog.

[23] Kawai K, Yamaga M, Iwamae Y (2004). A PLCδ1-binding protein, p122RhoGAP, is localized in focal adhesions. Biochem Soc Trans.

[24] Nabel EG, Gordon D, Yang ZY (1992). Gene transfer in vivo with DNA-liposome complexes: lack of autoimmunity and gonadal localization. Hum Gene Ther.

[25] Zhu N, Liggitt D, Liu Y, Debs R (1993). Systemic gene expression after intravenous DNA delivery into adult mice. Science.

[26] Chen QR, Kumar D, Stass SA, Mixson AJ (1999). Liposomes complexed to plasmids encoding angiostatin and endostatin inhibit breast cancer in nude mice. Cancer Res.

